# Unravelling the Effect of Citrate on the Features and Biocompatibility of Magnesium Phosphate-Based Bone Cements

**DOI:** 10.1021/acsbiomaterials.0c00983

**Published:** 2020-09-09

**Authors:** Rita Gelli, Gemma Di Pompo, Gabriela Graziani, Sofia Avnet, Nicola Baldini, Piero Baglioni, Francesca Ridi

**Affiliations:** † Department of Chemistry “Ugo Schiff” and CSGI, University of Florence, via della Lastruccia 3-13, 50019 Sesto Fiorentino, Italy; ‡ BST Biomedical Science and Technologies Lab, IRCCS Istituto Ortopedico Rizzoli, 40136 Bologna, Italy; § Department of Biomedical and Neuromotor Sciences, University of Bologna, 40127 Bologna, Italy; ▲ Laboratory of Nanobiotechnology (NaBi), IRCSS Istituto Ortopedico Rizzoli, via di Barbiano 1/10, 40136 Bologna, Italy

**Keywords:** magnesium phosphates, bone cements, citrate, bioactive materials, bone regeneration

## Abstract

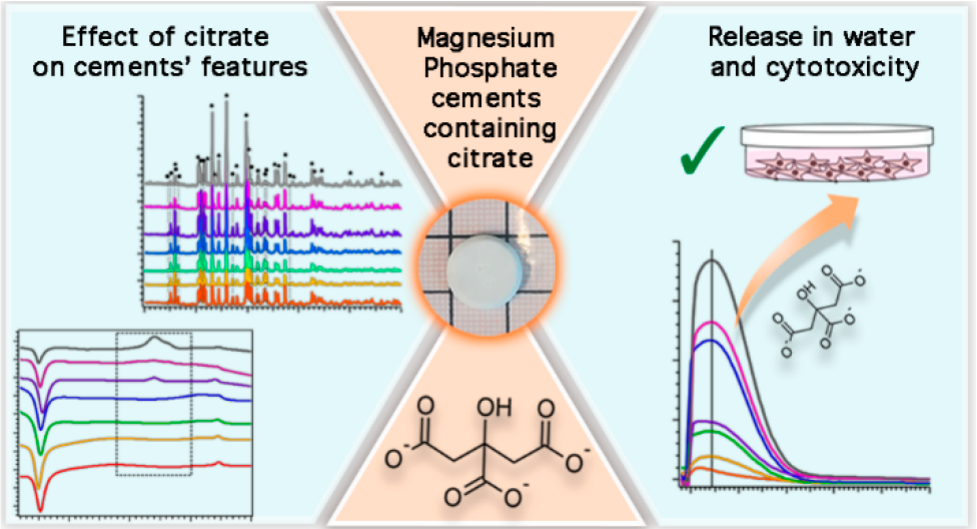

In the framework of new materials for orthopedic applications, Magnesium Phosphate-based Cements (MPCs) are currently the focus of active research in biomedicine, given their promising features; in this field, the loading of MPCs with active molecules to be released in the proximity of newly forming bone could represent an innovative approach to enhance the in vivo performances of the biomaterial. In this work, we describe the preparation and characterization of MPCs containing citrate, an ion naturally present in bone which presents beneficial effects when released in the proximity of newly forming bone tissue. The cements were characterized in terms of handling properties, setting time, mechanical properties, crystallinity, and microstructure, so as to unravel the effect of citrate concentration on the features of the material. Upon incubation in aqueous media, we demonstrated that citrate could be successfully released from the cements, while contributing to the alkalinization of the surroundings. The cytotoxicity of the materials toward human fibroblasts was also tested, revealing the importance of a fine modulation of released citrate to guarantee the biocompatibility of the material.

## 1 Introduction

The aging of the population and the associated increase in bone defects and diseases make the quest for new materials for bone regeneration a major societal challenge.^1^ Bone is a self-healing tissue which can heal small sized defects and restore its mechanical and biological functions; nevertheless, this process is only possible for fractures below a critical size,^2^ and several approaches have been proposed to support the natural bone regeneration process. Among the developed synthetic materials, bone cements hold an important position,^3,4^ being used as bone defect fillers in maxillofacial surgery, dentistry, orthopedic fracture treatments, and to augment bone weakened due to osteoporosis.^5,6^ They consist of a powder and a liquid component which react generating a paste that in time sets and forms a hardened cement, because of the entanglement of the newly formed crystals. The advantage of having a moldable and sometimes even injectable material able to harden directly in the body provides unique advantages over bioceramics, as it allows to perform minimally invasive surgeries (with benefits for the patient itself and for the hospitalization costs) and to have an intimate adaption to the surrounding bone, even for irregularly shaped cavities.^3^ Ideally, a bone cement should be biocompatible, set at physiological temperature in a time frame suitable for injection and surgery, and form a porous material with mechanical properties compatible with bone, being eventually resorbed by the newly formed tissue.^4^

The reason for the widespread investigation and use of calcium phosphate cements (CPCs) is related to their chemical similarity to bone inorganic matrix, which consists of poorly crystalline, calcium-deficient hydroxyapatite nanoplatelets.^7^ Given the great number of advantages associated with these types of materials, such as biocompatibility, bioactivity, and osteoconductivity,^3,8^ research is presently focusing on new strategies to further improve their performances and to overcome some of the drawbacks associated with their use.^9^ It was recently proposed that magnesium phosphate-based cements (MPCs) could be used as an alternative to CPCs. In addition to the increased mechanical strength,^10^ which is still not sufficient for load-bearing applications, MPCs have shown a more rapid dissolution rate in aqueous media, which results in enhanced in vivo resorption that favors bone growth;^11^ moreover, MPCs have shown intrinsic adhesive capabilities, which are not reported for CPCs.^9,12,13^ Another advantage of using MPCs over CPCs is that the in vivo release of Mg^2+^ was shown to stimulate osteoblast differentiation and to inhibit osteoclast formation, thereby favoring bone regeneration.^14,15^ Some MPCs were even reported to be naturally antibiotic,^10,16^ while retaining good biocompatibility both in vitro and in vivo.^2,17−19^ All these features disclose the great potentialities of MPCs, which are still underexplored in comparison to the well-established CPCs.^9^ The properties of MPCs, which are typically prepared by mixing MgO or Mg_3_(PO_4_)_2_ (trimagnesium phosphate, TMP) with aqueous solutions of phosphate salts, can be improved by the addition of modifiers, such as reaction retardants, pore formers, and radio-opaque agents; to the best of our knowledge, the inclusion of bioactive molecules in the cement matrix, as well as their release profile when in contact with biological fluids, has never been addressed so far.

In this work, the possibility of including citrate (C_6_H_5_O_7_^3–^) in MPC-based formulations was explored. This ion represents about 5 wt% of bone,^20^ where it bridges between mineral hydroxyapatite platelets^21^ and controls their size and crystallinity, acting as a key mineralization regulator.^22^ Because its p*K*_a_ values are 2.9, 4.3, and 5.6, in human tissues, it is mainly present in unprotonated form: ∼90 wt% is accumulated in bone, while the rest is included in physiological and pathological calcifications (dentin, kidney stones, and atherosclerotic plaques) and soft tissues (brain, prostate, and kidneys).^23^ The natural existence of citrate and its importance in bone physiology suggest that this ion should be taken into consideration in the design of orthopedic biomaterials and scaffolds.^24^ Its inclusion in bone biomaterials has demonstrated many interesting outcomes: a recent study showed that citrate, whether present on a biomaterial surface or supplemented into cell culture media, has unique effects on gene expression, as it upregulates Osterix (a transcription factor which is essential for osteoblast differentiation and bone formation) in myoblasts, and alkaline phosphatase (an enzyme which is among the first functional genes expressed in the process of calcification) in both myoblasts and mature osteoblasts.^24^ Citrate also has implications in the treatment of osteopenia and osteoporosis, pathological conditions that occur due to aging and result in decreased bone mineral density, deterioration of bone architecture, and ultimately, enhanced susceptibility to fractures.^25^ These pathologies can be enhanced by acidosis, a metabolic condition in which high acid load exceeds physiological neutralization capacity and which favors bone resorption processes and inhibits osteogenic functions.^26^ Therefore, citrate-based treatments that potentially neutralize acidosis might represent effective strategies for preventing the progression of osteopenia. In this context, clinical evidence has pointed out the beneficial effects of K-citrate supplementations on bone turnover in both healthy^27^ and osteopenic subjects.^28^ Furthermore, in vitro data have revealed that K-citrate significantly inhibits osteoclastogenesis and potentiates the antiosteoclastic activity of a standard bisphosphonate, by directly affecting bone cells regardless of its alkalinizing effect.^29^ In light of these studies, the integration of citrate in MPCs would represent an innovative and unexplored field with many potential outcomes. The preparation of TMP-based MPCs has been poorly investigated; in the only study found in the literature,^30^ cements are obtained using a combination of (NH_4_)_2_HPO_4_ and a citrate salt, where citrate is used as a viscosity modifier. As a consequence, the study is mainly focused on the rheological and mechanical properties of the material and reveals the beneficial role of citrate on the handling properties and the injectability of the cement. In contrast, citrate was often included in CPCs, with the main purpose of enhancing setting time and injectability of the pastes.^31−38^ Very recently, citric acid was included in the formulation of a mixed Ca–Mg system prepared with MgO, KH_2_P_2_O_4_, and Ca(H_2_PO_4_)_2_, and the results demonstrated that cements with satisfactory setting time, mechanical strength, and good cytocompatibility and osteoinductivity could be obtained.^39^ However, to date, the loading of citrate on bone cements as a therapeutic agent has never been explored.

The aim of this work is to develop an innovative MPC able to release citrate for applications in the orthopedic field. MPCs were prepared upon reaction of TMP powder with various aqueous solutions at different ratios between diammonium hydrogen phosphate (DAHP) and diammonium citrate (DAC). The effect of citrate on the features of both the pastes and the final cements was investigated by means of a multitechnique approach, unravelling the effect of this ion on the properties of the material. The incubation of the cements in water was then investigated, revealing the ability of all the formulations to release citrate in a concentration-dependent manner. In principle, the released citrate could have beneficial effects in the formation of new bone, while the MPC matrix could provide mechanical stability to the resorbing bone. The cytocompatibility of the prepared cements was finally assessed in vitro and compared to a CPC commonly used in orthopedics, revealing the beneficial effect of citrate toward cell viability.

## 2 Materials and Methods

### 2.1 Cement Preparation

#### 2.1.1 Preparation of Magnesium Phosphate-Based Bone Cements

Tri-Magnesium Phosphate (TMP, Mg_3_(PO_4_)_2_) powder was obtained by calcination of MgHPO_4_·3H_2_O (Newberyite, Aldrich, purity ≥97%) and Mg(OH)_2_ (Magnesium hydroxide, Fluka, purity ≥95%) in molar ratio 2:1.^30^ The two reactants were carefully mixed and placed in ceramic crucibles, which were heated in a muffle furnace at 1000 °C for 5 h. The calcined product was crushed with a mortar and pestle and sieved using a 150 μm sieve.

The cements were prepared by mixing TMP with different aqueous solutions at a powder to liquid (P/L) ratio of 2 g/mL. Seven solutions at different contents of diammonium hydrogen phosphate (DAHP, (NH_4_)_2_HPO_4_, Riedel de Haën, purity ≥99%) and dibasic ammonium citrate monohydrate (DAC, HOCCOOH(CH_2_COONH_4_)_2_·H_2_O, Carlo Erba, purity ≥99%) were used, and their composition is reported in Table 1. Salts were dissolved in deionized water. The prepared volume was 1 mL, with a total salt concentration (DAHP + DAC) of 3.500 M. The amount of TMP used to prepare the disks depends on the conducted experiment: for the Gillmore test, pastes were prepared with 0.5 g of TMP (+250 μL of solution), whereas for all the other analyses samples were prepared with 0.3 g of TMP (+ 150 μL of solution). In all cases, the two components were mixed for 30 s and the resulting pastes were placed into silicon cylindrical molds (⌀ =1 cm) and set at 37 °C and relative humidity (RH) > 96% for 5 days. The samples are named after the solution used for their preparation (for instance, A is the sample prepared with solution A).

**Table 1:** Composition of the Solutions Used to Prepare the Cements

sample	DAC [M]	DAHP [M]	DAC [g]	DAHP [g]	molar ratio DAC/DAHP
A		3.500		0.4620	
B	0.025	3.475	0.0061	0.4589	0.007
C	0.050	3.450	0.0122	0.4556	0.014
D	0.100	3.400	0.0244	0.4490	0.029
E	0.500	3.000	0.1221	0.3962	0.167
F	1.000	2.500	0.2442	0.3302	0.400
G	2.000	1.500	0.4884	0.1981	1.330

#### 2.1.2 Preparation of Tricalcium Phosphate-Based Cements

The α-tricalcium phosphate-based cements (TCP) were prepared as previously described.^40^ Briefly, TCP was obtained by heating in a furnace (Hobersal CNR-58), in air, an appropriate mixture of calcium hydrogen phosphate (CaHPO_4_, Sigma–Aldrich) and calcium carbonate (CaCO_3_, Sigma–Aldrich) at 1400 °C for 2 h, followed by quenching in air. The TCP powder was then first milled with 10 balls (*d* = 30 mm) for 60 min at 450 rpm followed by a second milling for 70 min at 500 rpm with 100 balls (*d* = 10 mm). Precipitated hydroxyapatite (2 wt %; Alco) was added as a seed in the powder. The cement’s liquid phase consisted of an aqueous solution of 2.5 wt % disodium hydrogen phosphate (Na_2_HPO_4_, Panreac). A powder to liquid ratio of 2.86 g/mL was used to prepare disks of 14 mm diameter and 0.25 mm high in Teflon molds. The cements were allowed to set in Ringer’s solution (0.9 wt % NaCl) for 7 days at 37 °C to obtain the calcium deficient HA.

### 2.2 Cement Characterization

#### 2.2.1 Setting Time

The initial and final setting times of the pastes were measured using a Gillmore apparatus (Matest), according to the ASTM standard C-266. This experiment was carried out on fresh cements, prepared by mixing 0.50 g of TMP with 250 μL of solution for 30 s. The pastes were immediately placed in plastic molds; the setting occurred in controlled temperature and humidity conditions (*T* = 37 °C, RH > 96%) and, every 2 min, samples were removed from the incubation chamber and tested with the Gillmore apparatus. The cement is considered to have attained its initial or final setting time when its surface respectively bears the initial or final Gillmore needle without appreciable indentation (initial needle ⌀ = 2.12 mm, weight 113 g, and final needle ⌀ =1.06 mm, weight 453.6 g). Three replicates of each sample have been analyzed.

#### 2.2.2 X-ray Diffraction

X-ray Diffraction (XRD) patterns were collected using an X-ray Diffractometer D8 Advance with a DAVINCI design (Bruker). A small portion of the set cements was ground using a mortar and pestle and flattened on a zero-background sample holder. Diffraction data were collected in the 2θ range from 10° to 60°, with an increment of 0.03° and 0.3 s per step, using a 0.6 mm slit. Peaks assignment was based on the Powder Diffraction Files (PDF) of the International Centre for Diffraction Data (PDF: 25–1373 for farringtonite and 03–0240 for struvite). The relative amount of the formed phases was estimated by means of the Rietveld method, using the software Topas (Bruker). The CIF (Crystallographic Information File) data used for the analysis were obtained from the American Mineralogist Crystal Structure Database (0011901 for farringtonite and 0009807 for struvite). The percentage error associated to the fitting is 10%.

#### 2.2.3 Mechanical Properties

The compressive strength of the set cements was tested by performing a compression analysis with an electromechanical universal testing machine Instrom 5500 L with a 10 kN load cell. The samples were prepared by mixing 1.8 g of TMP with 0.900 mL of DAHP solution 3.5 M; the pastes were poured in cylindrical molds (diameter 9 mm, height 2.3 cm) and were set at 37 °C, at relative humidity >90% for 3 days. The specimens were extracted from the molds and polished with abrasive paper so to obtain an aspect ratio of about 2 (height/diameter) and to make the two surfaces of the cylinders as flat as possible. Four samples were prepared for each composition, and the reported results refer to the average value ± the associated standard deviation.

#### 2.2.4 Field Emission-Scanning Electron Microscopy

The morphology and the microstructure of the samples were investigated using Field Emission-Scanning Electron Microscopy (FE-SEM). Cross sections of the cements were fixed on aluminum stubs by means of conductive tape. The measurements were performed by means of a Zeiss ΣIGMA FE-SEM (Carl Zeiss Microscopy GmbH), with an accelerating voltage of 2.0 kV and a sample–detector distance ∼2 mm.

#### 2.2.5 Thermal Analysis

Thermal analysis on the cements was carried out using a Simultaneous Thermogravimetry/Differential Scanning Calorimetry (TGA/DSC) SDT Q600 from TA Instruments. Ground samples were placed in an alumina pan and measurements were conducted in N_2_ atmosphere (flow rate 100 mL/min) from room temperature to 1000 °C, with a ramp of 10 °C/min. For comparison, a mixture of TMP/water (prepared with 0.3 g of TMP and 150 μL of water and aged for 5 days) and an aliquot of DAC were also analyzed.

### 2.3 Incubation of Cements in Water

Cement samples of 1 cm diameter and ∼4 mm thickness were placed in a cell culture 48-well plate and incubated with 1 mL of water at 37 °C for 4 days, in conditions analogous to those used to obtain the extracts (see section 2.4.1). After the incubation of the cements, the resulting solutions were used for monitoring both the pH and the released citrate, as described below.

The pH was daily monitored by means of a S2K922 pH meter (Isfetcom Co., Ltd.). The weight % at the end of the experiment was calculated as the ratio % between the initial and final weight of the dried cements (after incubation, samples were dried at 37 °C for 7 days).

The quantification of released citrate was conducted by means of a spectrophotometric method reported in the literature.^41^ The calibration curve was obtained analyzing aqueous solutions with a known citrate concentration (prepared using serial dilutions at DAC 10 mM, 8 mM, 6 mM, 4 mM, 2 mM, and 1 mM), and diluting them with HCl 0.25 M v/v 1:1. As reference for the UV spectra, a sample water/HCl v/v 1:1 was used. The obtained spectra are reported in Figure S1a in the Supporting Information (SI), whereas the calibration line (*y* = 0.201*x* + 0.005, *R*^2^ = 0.999) is shown in Figure S1b. 200 μL of solution resulting from the cement incubation or of standard solution +200 μL HCl 0.25 M were mixed in micro quartz cuvettes of 1 cm-optical path length. For samples E, F, and G, 20 μL of samples + 380 μL of HCl were used to decrease absorbance. Three replicas for each sample were analyzed. Measurements were carried out on a UV–vis spectrophotometer Cary3500 (Agilent) in the range 300–190 nm, integration time 1 s, *T* = 25 °C, and bandwidth 1 nm.

### 2.4 Cytotoxicity Experiments

#### 2.4.1 Preparation of the Cement Extracts

To prepare the extracts of the magnesium phosphate-based cements, cement samples of 1 cm diameter and ∼4 mm thickness were placed into a 48-well plate and incubated with 1 mL of Iscove’s modified Dulbecco’s medium (IMDM) supplemented with 10% heat-inactivated fetal bovine serum (FBS, Euroclone), plus 100 units/mL penicillin, and 0.1 mg/mL streptomycin (both from Life Technologies) (complete IMDM). After 4 days of incubation at 37 °C in a humidified atmosphere containing 5% CO_2_, each supernatant (cement extract) was collected and centrifuged (13 000 rpm, 20 min) to be used for the cell viability assay. The extract of the conventional phosphate-based cement (TCP) was prepared by the same protocol to be used as reference material.

#### 2.4.2 Cell Viability Assay

The cytotoxic effect of the cement extracts was evaluated on human MRC-5 fibroblasts (American Type Culture Collection, ATCC; TIB-71) using the Alamar blue test. The Alamar blue assay is based on the metabolic ability of viable cells to reduce resazurin to resorufin, a highly fluorescent compound. Briefly, cells were seeded into 96-well plates (2.5 × 10^3^ cells/well) in complete IMDM and maintained at 37 °C in a humidified atmosphere containing 5% CO_2_. After cell adhesion, the medium was discarded, and the cells were incubated with the cement extracts.

After 24, 48, and 72 h, the culture medium was discarded, cells were washed with PBS, and fresh medium containing 10% of Alamar Blue (Invitrogen) was added to the culture. The same solution was placed in an empty well and used to detect the background fluorescence (blank). The plates were incubated at 37 °C in a humidified atmosphere of 5% CO_2_ for 4 h. After incubation, the cell culture supernatants were transferred to a new plate, and the fluorescence was measured by a microplate reader (Tecan Infinite F200pro) using an excitation and emission wavelength of 540 and 590 nm, respectively. Data were expressed as Relative Fluorescence Units (RFU) after blank subtraction.

### 2.5 Statistical Analysis

Because of the small number of observations, data were not considered normally distributed and therefore nonparametric tests were used. Statistical analysis was performed using the StatView 5.0.1 software (SAS Institute, Inc.). For the difference between two groups, the Mann–Whitney U test was used. Data were expressed as mean ± standard error (SEM), and only *p*-values <0.05 were considered for statistical significance.

## 3 Results

### 3.1 Characterization of the Pastes and of the Set Cements

**Figure 1:**
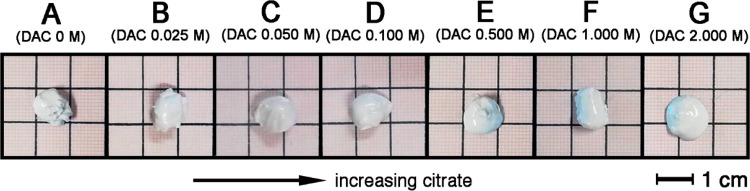
Photos of the pastes after 1 min from the mixing; the content of citrate in the formulations increases from left to right.

The initial consistency and the handling properties of the pastes were evaluated by observing their appearance immediately after mixing (see Figure 1): when TMP powder (characterization reported elsewhere^42^) is mixed with DAHP and DAC-based solutions, an easily moldable paste quickly forms. As the citrate content in the formulation increases, pastes appear more uniform and less viscous, displaying improved handling properties. The observed liquefying effect of citrate in similar formulations was already described in the literature in the work by Moseke et al.;^30^ while for CPCs this effect is ascribed to an increased dispersibility of the initial TMP particle agglomerates due to the greater electrostatic repulsion given by citrate adsorption,^31^ for MPCs this effect is reported to be caused by the adsorption of citrate ions on the particles during the setting reaction.^30^

**Figure 2:**
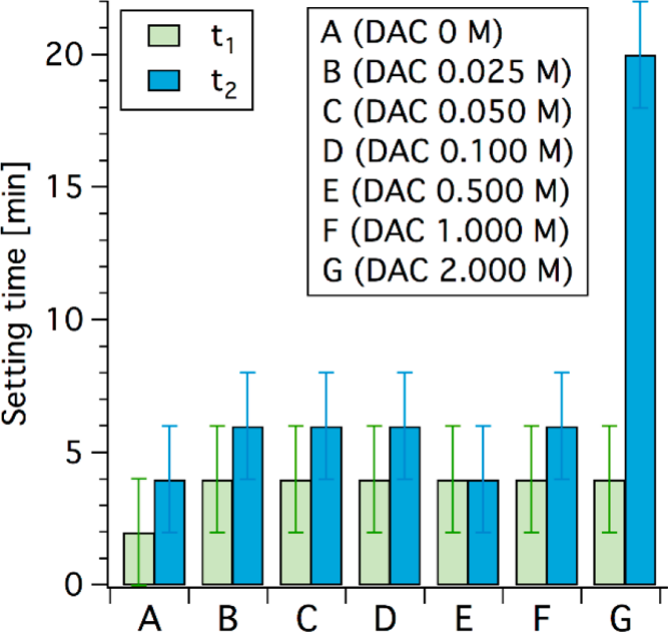
Initial (*t*_1_) and final (*t*_2_) setting times of the pastes measured by means of the Gillmore test (error bars: ± 2 min).

The setting time of the pastes was quantitatively evaluated by means of the Gillmore test, and the obtained results are shown in Figure 2.

All the investigated samples attain the initial setting time (*t*_1_) in a few minutes, irrespective of the amount of citrate present in the aqueous solution used to prepare them. However, the final setting time (*t*_2_) appears to be related to the citrate content, given that sample G, which is prepared with the solution at highest DAC concentration (see Table 1), has a *t*_2_ of ∼20 min, while the final setting of the other samples occurs within 6 min. These findings are in agreement with literature reports on citrate-based MPCs, which show that an increase in citrate content prolongs the setting time of the cements.^30^ According to clinical requirements, bone cements should display 3 ≤ *t*_1_ < 8 min and *t*_2_ ≤ 15 min,^4^ samples from A to F show promising features.

**Figure 3:**
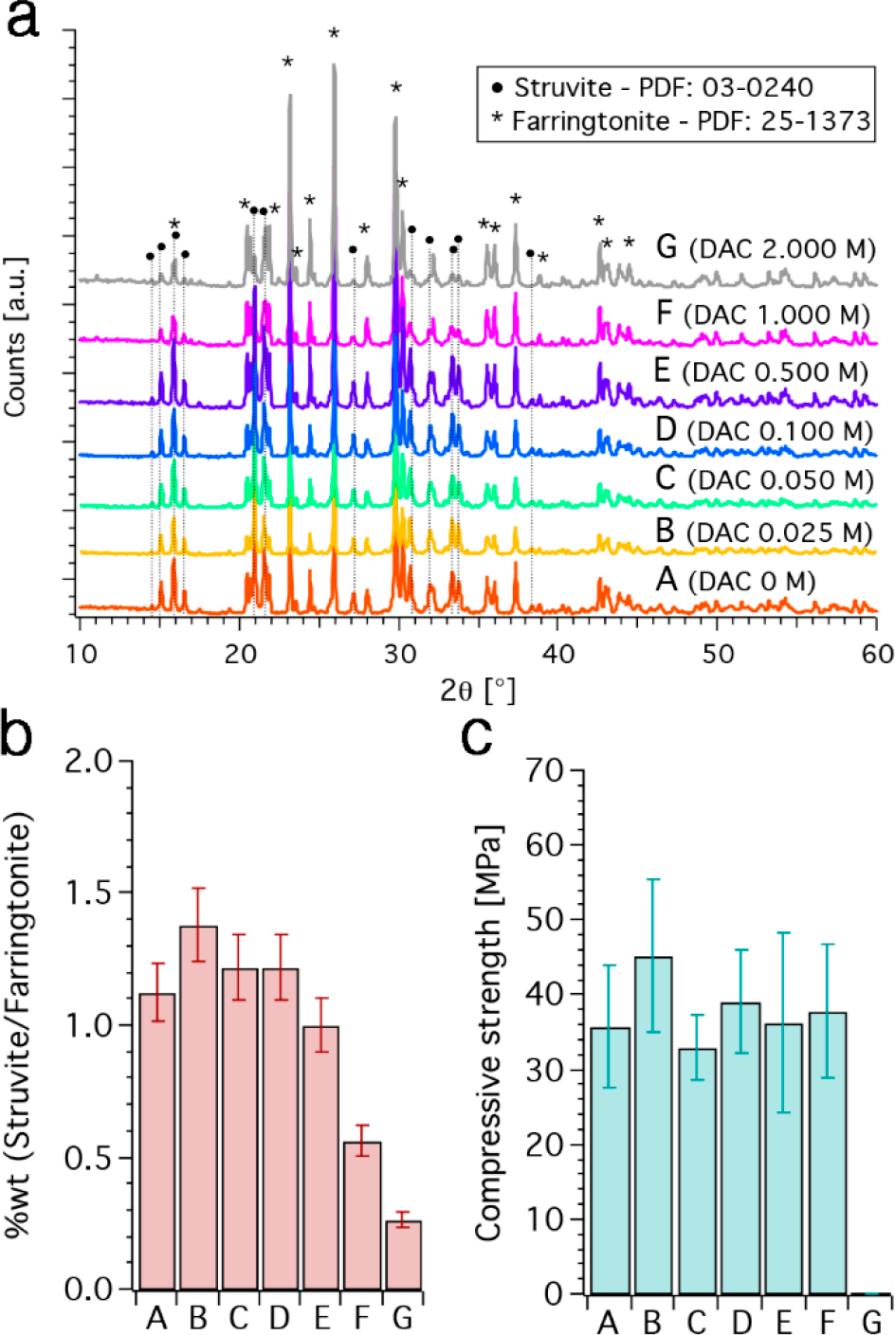
(a) XRD diffractograms of the investigated samples. The peaks diagnostic of struvite (PDF: 03–0240) are indicated by the dashed lines and the dots, whereas farringtonite (TMP, PDF: 25–1373) signals are denoted with a star. The diffractograms are offset for display purposes; (b) ratio between struvite and farringtonite, obtained by means of a Rietveld fitting on the diffractograms. As the contribution of a hypothetical amorphous phase due to citrate was not included in the fitting, we only report the relative ratio between the obtained wt% of struvite vs farringtonite; and (c) compressive strength results (average of 4 measurements per sample ± standard deviation). The value for G is close to 0 (0.006 ± 0.001 MPa).

After setting, all cements result in hard and compact materials (see a representative picture in Figure S2). The nature of the crystalline phases formed upon setting was analyzed by means of XRD, and the obtained results are reported in Figure 3a. All samples display signals compatible with the simultaneous presence of farringtonite (mineral name of TMP, indicated by the stars in the diffractogram) and struvite (MgNH_4_PO_4_·6H_2_O, see the dots in Figure 3a); the former is due to unreacted TMP, while the latter is the binding phase that, according to the literature, forms upon reaction of TMP and DAHP.^30^ From a qualitative point of view, the comparison of the relative intensity of struvite diagnostic peaks with the TMP ones suggests that the relative amount of struvite in the cements decreases as the DAC content increases (i.e., DAHP content decreases). This is evident from the direct comparison of the pattern of sample A (no citrate) and G (highest citrate content), which is reported in Figure S3. It also appears that the use of DAC over DAHP does not lead to the formation of a new crystalline phase, as no new peak can be detected in the diffractograms of samples from B to G; this suggests that citrate ions might be included in the final cements as components of an amorphous phase. A quantitative indication of the relative amount of struvite with respect to farringtonite can be obtained by fitting the experimental diffractograms by means of the Rietveld method. The results, reported in Figure 2b, confirm the conducted observations, as the amount of struvite decreases with the increase of DAC present in the formulation.

The compressive strength of the cements was also tested, being a feature of paramount importance for this type of material.^3,9^ The results, reported in Figure 3c, show that all compositions, except for G, give rise to cements with satisfying compressive strength values, which range from about 30 to 45 MPa, in line with typical values for MPCs.^9^ The inclusion of moderate citrate amounts in the formulations (up to 1 M for sample F) does not hamper the compressive strength of the cements, while formulation G (DAC 2 M) displays very poor mechanical properties due to the limited struvite network formed, consistent with the literature.^30^

**Figure 4:**
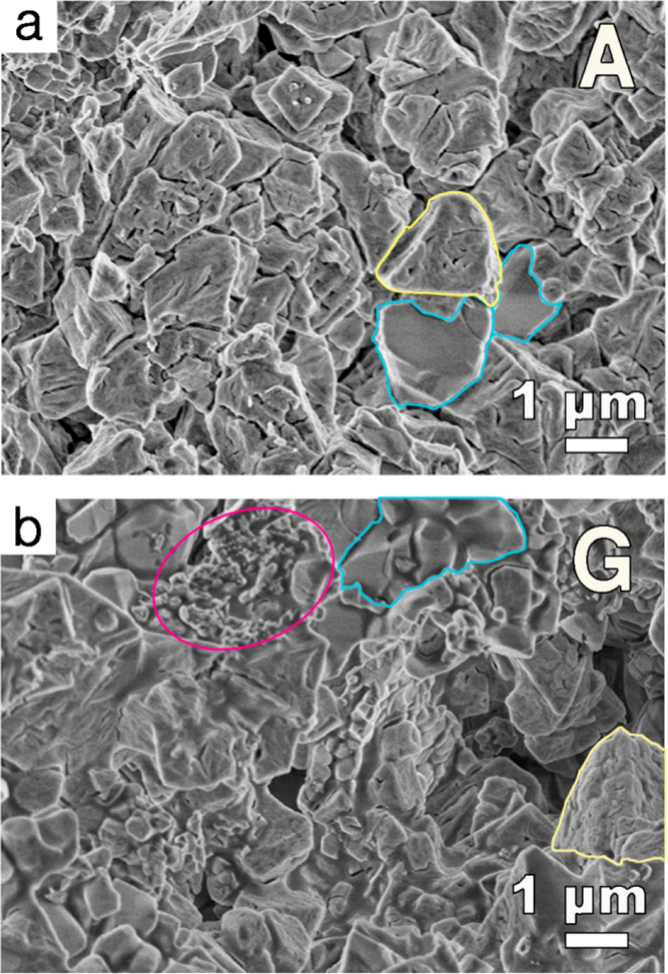
FE-SEM micrographs of cross sections of cements (a) A (DAC 0 M) and (b) G (DAC 2.000 M). The yellow areas delimit the structures typical of struvite, the blue ones highlight TMP crystals, whereas the pink ones refer to structures present only in citrate-containing samples.

The morphology and the microstructure of the cements were investigated by means of FE-SEM. Figure 4 shows the morphology of a cross-section of sample A and G, which display major differences, while the micrographs referred to all the samples are reported in Figure S4. Sample A is rich in struvite crystals, which are characterized by a rough surface with inner cracks and porosities^42^ (see as an example the yellow highlighted area in Figure 4a). A limited number of TMP grains with a smooth surface, which are outlined by the blue regions in the figure, is also visible. Increasing the DAC/DAHP ratio in the samples leads to a reduction of the extent of struvite network, as we can clearly observe that the amount of objects with the typical struvite morphology decreases from sample A to G (see Figure S4), in agreement with the smaller conversion to struvite due to the reduced DAHP amount (i.e., higher DAC amount). A large number of unreacted TMP grains is also visible, consistently with the XRD results previously presented; moreover, some structures with an irregular and undefined shape are detectable (see the pink ellipse in Figure 4b), which can be related to the presence of citrate, possibly resulting from the reaction of TMP with DAC in some regions of the inorganic crystals.

**Figure 5:**
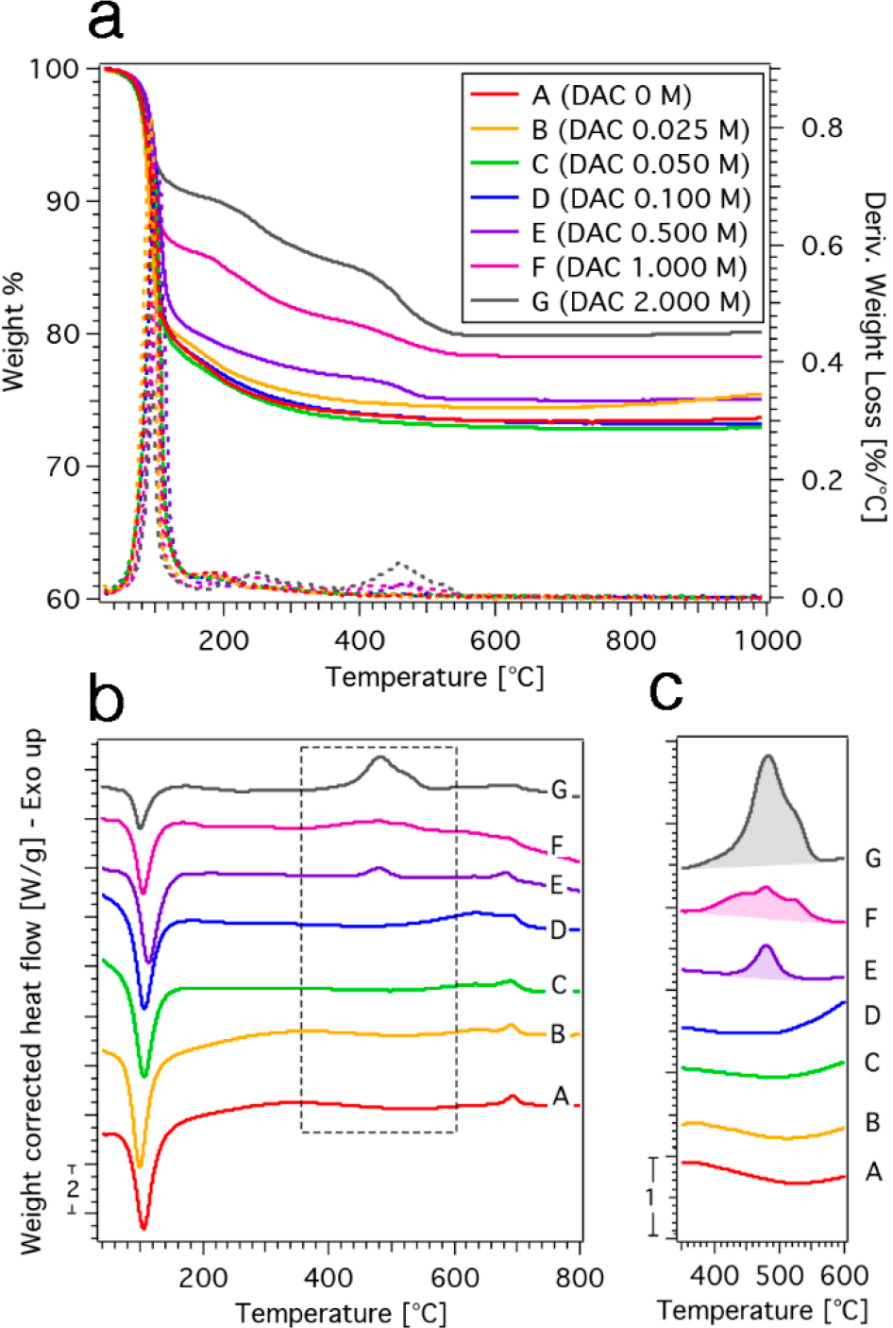
(a) Thermogravimetric curves (solid lines, left axis) and derivative curves (dashed lines, right axis) of the investigated cements; (b) heat flow profiles acquired simultaneously to the TGA measurements; and (c) zoom of the region 350–600 °C, with the linear integration of the exothermic peak centered at ∼480 °C. The curves are offset for display purposes.

In order to gain more insights into the phases present in the cements and their features, the samples were analyzed by means of thermogravimetry coupled with differential scanning calorimetry, allowing for the simultaneous determination of the degradation profile and the phase transitions which occur in the samples upon heating. The weight % as a function of temperature and the derivative curves (dashed lines) are reported in Figure 5a, while the heat flow profiles are shown in Figure 5b.

The weight loss profile of sample A is entirely ascribable to the thermal decomposition of struvite (TMP, when heated up to 1000 °C, loses about 0.2% of its weight, data not shown). The steep weight loss up to 150 °C, which is of 21 wt%, is associated to the loss of 5 mol of crystallization water, with the formation of dittmarite (MgNH_4_PO_4_·H_2_O);^43−46^ the subsequent weight loss is due to the loss of 1 mol of crystallization water and ammonia, which leads to amorphous MgHPO_4_ (200–550 °C). Considering that, at 1000 °C, a pure struvite sample would lose ∼55% of its initial weight,^44^ while sample A displays a weight loss of 27%, the composition of our cement can be roughly estimated (∼49% of struvite and ∼51% of TMP, in good agreement with the results obtained with the Rietveld fitting of the XRD data, i.e., ∼53% of struvite and ∼47% of TMP). The thermograms of samples containing a low amount of citrate (B, C, and D) do not show significant differences in the degradation profile with respect to sample A. Conversely, for samples E, F, and G, as the amount of DAC in the formulation increases, the extent of the weight loss below 150 °C diminishes (20% for sample A, 14% for sample B, and 9% for sample D). This finding is consistent with the decrease in struvite content observed by means of both XRD and SEM analyses. Moreover, the DTG profiles of these samples show new small peaks in the region 200–300 °C and 350–550 °C, whose intensity increases consistently with the amount of citrate in the formulation: these weight losses, that do not occur in the sample prepared only with DAHP, are reasonably due to the new amorphous phase that forms by the reaction of TMP and DAC. The analysis of the heat flow profiles reveals some interesting aspects (see Figure 5b). All samples show an endothermic peak at ∼100 °C, which is associated to the loss of the crystallization water previously discussed. Samples A–E display small exothermic peaks at ∼670 °C, due to the conversion of MgHPO_4_ (formed by heating struvite above 200 °C) to Mg_2_P_2_O_7_.^43,44^ More interestingly, samples E, F, and G display an exothermic peak with a maximum at ∼480 °C, whose area grows accordingly to the amount of citrate in the formulations (Δ*H* = 84 J/g for E, 220 J/g for F, and 559 J/g for G). It is hypothesized that the exothermic event which generates this peak is the crystallization of the amorphous phase formed in citrate-containing samples, given that amorphous phases are often reported to crystallize upon heating.^47,48^ To further support the evidence that this peak is due to a new amorphous binding phase formed upon the interaction of citrate with the inorganic phase, a sample made of TMP/water and a sample of pure DAC (see section 2.2.5) were analyzed: results reported in Figure S5 show that these samples do not display such a peak in the heat flow profile, demonstrating that it is diagnostic of a new phase which forms only when citrate interacts and reacts with TMP.

### 3.2 Incubation in Water and Release of Citrate

**Figure 6:**
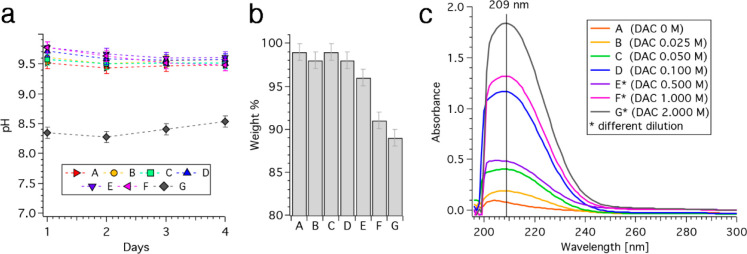
(a) pH vs time of the incubating solutions (error bars: ± 0.1); (b) weight % of the dried cements compared to their weight before incubation; (c) UV spectra of incubating solution, treated as described in section 2.3 (sample A, B, C, and D: 200 μL of samples + 200 μL HCl, sample E, F, and G: 20 μL + 380 μL HCl).

The ability of the cements to release citrate was assessed by incubating them in water, as described in section 2.3. The pH was daily monitored (see Figure 6a), revealing that all the formulations display an alkalinizing effect, as they lead to an increase in the pH of the medium in which they are incubated (pH of the used Milli-Q water before immersion of the cements: 6.5 ± 0.1). Formulations A–F provoke a rise in pH up to ∼9.5, which is constant throughout the incubation period; sample G, which has the highest citrate content, displays a milder effect, as the pH of its incubating solution does not exceed 8.6. This evidence suggests that struvite itself displays an alkalinizing effect, which is a promising feature in view of their application as bone cements (see the Discussion section).

At the end of the incubation period, cements were dried, and the comparison of their weight with the initial one is reported as weight % in Figure 6b. Despite all samples exhibiting a good stability against dissolution, the weight % of samples E, F, and G is lower than that of samples prepared with a smaller citrate amount; as bone cements should be able to gradually dissolve and be degraded when implanted in vivo, the modulation of citrate in the formulation could therefore be regarded as a strategy to enhance the dissolution process.

The aqueous media in which the cements were incubated were finally analyzed to understand if the citrate included in the formulations was effectively released upon contact with the aqueous solutions. The UV absorption spectra of samples treated as described in section 2.3 are reported in Figure 6c, and the presence of a peak centered at 209 nm reveals that all cements are able to release citrate, in a concentration-dependent fashion. The spectrum of sample A (orange curve in Figure 6c), which does not contain citrate, consists of a flat signal, proving that citrate is the only component that contributes to the absorbance in that spectral region. The concentration of citrate released in the solution was calculated according to the calibration line in Figure S1b, and it is reported in Table 2 together with the % of released citrate with respect to the amount present in the formulation.

**Table 2:** Released Citrate (as a Concentration, and as a Percentage with Respect to the Amount Present in the Formulation) in the Incubating Solution, As Calculated from the UV Spectra after 4 Days of Incubation

sample	citrate concentration [mM]	% released citrate
A		
B	2.0 ± 0.2	53 ± 6
C	4.0 ± 0.5	53 ± 6
D	12 ± 1.4	80 ± 10
E	39 ± 5	52 ± 6
F	125 ± 15	83 ± 10
G	211 ± 25	70 ± 8

### 3.3 Cytotoxicity Experiments

**Figure 7:**
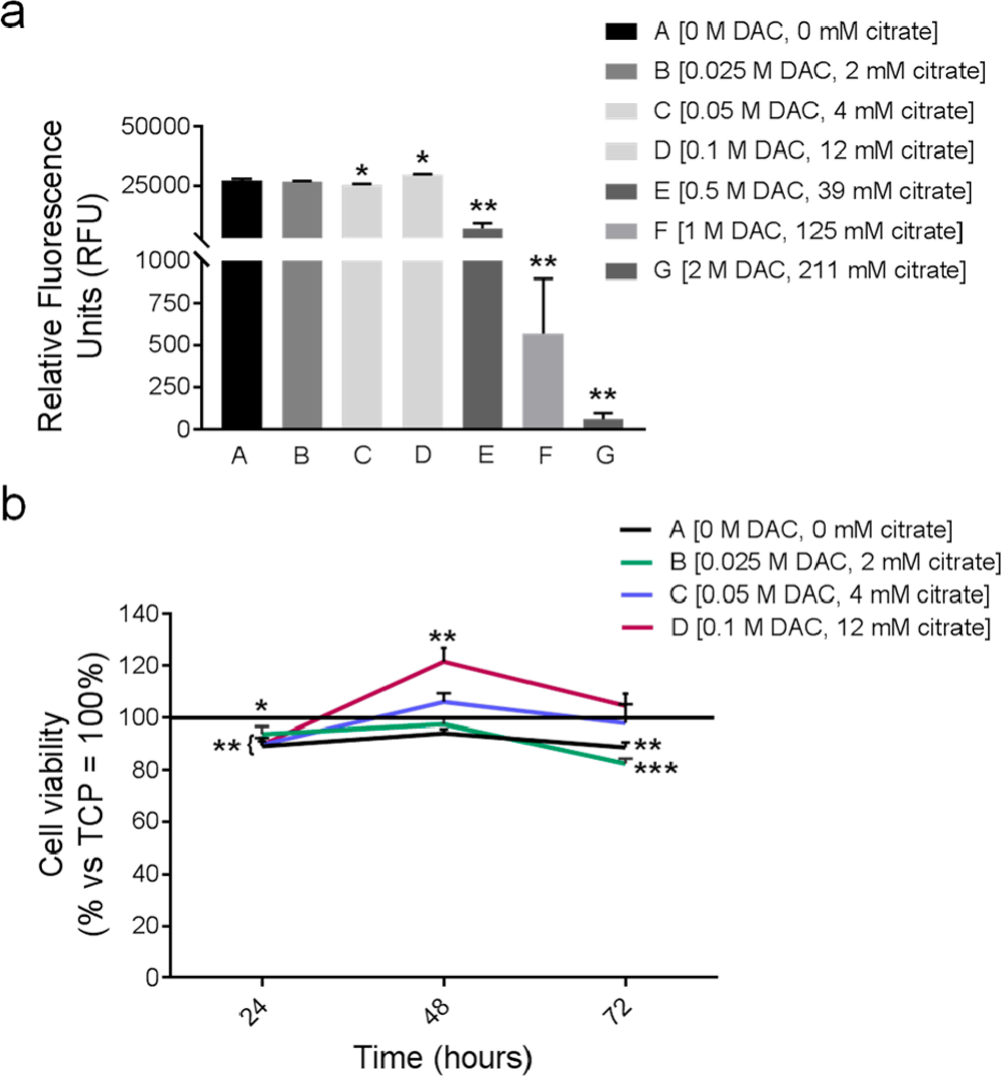
Alamar blue assay to evaluate the cytotoxicity of cement extracts on fibroblasts: (a) prescreening of extracts obtained from cements containing increasing DAC concentrations and releasing different amounts of citrate (within a large range). Data were expressed as Relative Fluorescence Units (RFU). The experiment was repeated twice in quadruplicate (*n* = 8). Mean ± SE, **p < 0.01, and *p< 0.05 vs extract from cement A [0 M DAC and 0 mM released citrate]; (b) cytotoxicity curve after 24, 48, and 72 h of cell exposure to selected nontoxic cement extracts. Data were expressed as percentage of cell viability with respect to TCP extract (=100%, bold line). The experiment was repeated twice in quadruplicate (*n* = 8). Mean ± SE, *** *p*< 0.001, ** *p*< 0.01, and * *p*< 0.05 vs TCP.

In order to assess the short-term cytotoxicity of the cements containing increasing concentrations of DAC (0, 0.025, 0.050, 0.1, 0.5, 1, and 2 M) and releasing a different amount of citrate (see Table 2), we exposed human fibroblasts to the cement extracts for 24 h. The cements containing the highest DAC concentrations, equal to or greater than 0.5 M, and that released into the extract more than 12 mM citrate, were significantly cytotoxic (more than 95% of inhibition, Figure 7a, *P* = 0.004 for E, F, and G vs the negative control A that corresponds to extract obtained from the cement not containing DAC).

The cement extract C only slightly affected cell viability (Figure 7a, *p* = 0.0379), whereas the cement extracts B and D were not toxic at all (for D, we observed an induction rather than an inhibition, *p*= 0.0148). To more deeply dissect the biological effect of nontoxic citrate concentrations (from 0 to 12 mM) in the cement extracts, the viability assay was repeated until 72 h. A conventional phosphate-based cement (TCP) was used as a reference. All the cement extracts, with or without citrate, only slightly impaired (about 10%) the cell viability compared to TCP extract at 24 h (*p* = 0.0038 for A, *p* = 0.0247 for B, and *p* = 0.0011 for C and D), and were completely not toxic at 48 h (Figure 7b). Notably and unexpectedly, at this end-point, the extract obtained from the cement containing the highest concentration of DAC and releasing the greater amount of citrate (cement D) was significantly less cytotoxic than the TCP extract (*p* = 0.0046). After 72 h, similarly to TCP extract, among the different cement extracts assayed, only C and D were not toxic.

## 4 Discussion

The development of new materials for bone regeneration is an active research topic and the interest toward magnesium phosphate-based systems is currently increasing.^2,9^ The advantage of magnesium phosphates with respect to their calcium counterpart relies on the higher solubility which corresponds to an enhanced in vivo resorbability, and on the effect of the released Mg^2+^ ions which are able to modulate osteoblasts and osteoclasts activity, thus favoring bone growth.^14,15^ The performances of pure inorganic systems can be improved by the incorporation of biorelevant molecules or macromolecules, which can either enhance the rheological properties of the material or the biological response of cells. The inclusion of citrate in MPCs could act on both aspects, as preliminary reports highlight its ability to improve the injectability and the rheological properties of the pastes^30^ and its beneficial action in bone regeneration processes.^27−29,49^ Nevertheless, the precise characterization of the type of phase formed by citrate in TMP-based cements and the biological response to such materials were never addressed so far. The preparation and characterization of systems containing different DAC/DAHP ratios allowed us to obtain a detailed picture of the effect of citrate on the cements: the results show that, in agreement with the literature,^30^ citrate can be used to improve their handling properties, as it makes the pastes less viscous and favors their moldability, and to adjust and extend the setting time of the pastes only when present above a certain amount. In fact, samples from A to F show similar values of setting times, likely due to a similar amount of formed struvite. The combined characterization of the set cements by means of XRD, FE-SEM, and thermal analysis allowed us to gain insights into the type of phases present in the cement: it is known from the literature that TMP and DAHP react forming struvite as a binding phase,^42^ whereas the nature of citrate inclusion in the cement matrix is not known. In particular, the goal of this investigation was to understand if this ion is included in the inorganic matrix as an unreacted material or if it can react with TMP, thus forming a new binding phase.

The obtained results show that the amount of struvite decreases with the decrease of DAHP present in the formulation, revealing that DAC does not take part to the formation of this binding phase. Nevertheless, we hypothesize that citrate is included in the cement matrix as an amorphous phase, given the crystallization peak observed in the heat flow profiles of samples prepared with a high DAC amount (Figure 5b,c). This amorphous binding phase is possibly more soluble than struvite and is responsible for the higher dissolution of samples rich in citrate after incubation in aqueous media (see Figure 6b). It is worth commenting that, in general, cements should not instantly dissolve upon contact with biological fluids, as their role is to provide mechanical support in the site where they are implanted; nevertheless, in time, they should progressively dissolve and be resorbed by cells, leaving space to the newly forming bone. The inclusion of citrate improves the dissolution rate of the cements, making the material closer to clinical needs. Moreover, because in vivo the resorption process is triggered by both passive dissolution, due to the solubility of the materials, and active dissolution by bone resorbing osteoclasts,^2^ the in vivo resorbability of these materials is expected to be improved.

In addition to the rate of dissolution of the cements, their incubation in water revealed two important aspects, namely their alkalinization ability and the effective release of citrate. The observation that all the investigated formulations contribute to increase the pH of the medium in which they are immersed is very promising in view of their application: given that bone pathologies such as osteopenia and osteoporosis are often related to interstitial/extracellular acidosis,^26^ the use of a material which is able to alkalinize the surroundings of the implantation site could attenuate this situation, preventing the progression of acidosis and the associated bone loss. It is worth commenting that the capability of alkalinizing the surrounding environment, for instance, is regarded among the main advantages of bioactive glasses as it is attributed to an antibacterial effect,^50^ but is not mentioned for calcium phosphates-based cements. As a consequence, this aspect could be significant in pushing toward a more extensive use of MPCs.

Results also demonstrate that a part of the citrate included in the formulation can be successfully released in aqueous media due to the dissolution of the matrix: this evidence is of utmost importance, given that the release of citrate would be essential for the ion to fulfill its beneficial action in bone regeneration processes. The concentration of released citrate is directly proportional to the amount used in the formulation of the material, therefore revealing a strategy to tune this feature.

From a biological point of view, the incorporation of citrate in bone cements is gaining an increasing interest in the field of orthopedic engineering due to its natural role in bone physiology. Citrate is an integral component of the bone tissue, and serves to maintain the integrity of the skeletal nano- and microstructures. It is synthesized by osteoblasts and, at the same time, influences their differentiation and functionality, thereby mediating the mineralization process.^51^ Accordingly, citrate incorporation into scaffolds intended for bone tissue engineering may favor their osteoinductive and osteoconductive properties.^23,52^ Nevertheless, to achieve this goal, a fine modulation of citrate release from the material is crucial since overconcentrations of citrate might be toxic for cells, thereby compromising the biocompatibility of the cement. Indeed, previous authors demonstrated that citrate concentrations above 11 mM produce a 50% drop in viability of 3T3 fibroblasts and MG63 osteoblast-like cells.^53^

The adjustment of the extracellular concentrations of citrate acquires further importance when considering its chelating properties. As an anion, citrate serves as a well-known sequestering agent of metal ions, including Ca^2+^ and Mg^2+^.^54^

The calcium-chelation potential of citrate is widely recognized in clinical practice^55^ and is exploited in blood sampling, hemofiltration, and hemodialysis due to its ability to inhibit the clotting cascade,^56^ and in the prevention of kidney stones formation by the binding to calcium oxalate crystals in the urinary tract.^57^

Despite the clinical benefits of citrate as sequestering agent, hypercitratemia can provoke symptomatic hypocalcemia and more rarely hypomagnesemia, with serious pathological sequelae such as coagulopathy, cardiac arrhythmias, and neuromuscular signs.^57,58^ Large citrate loads also can cause metabolic alkalosis as a result of hepatic metabolism of citrate to bicarbonate.^59^

Of note, the chelating properties of citrate might pose a threat to the bone microenvironment due to the relevant active role of magnesium and calcium ions in the biology and functions of both osteoblasts^14,15,60^ and osteoclasts.^61,62^

To evaluate the feasibility of using MPC-citrate based cements in the clinical setting, as a first step, their cytotoxicity on human cells was assessed. To this aim, human fibroblasts were exposed in vitro to the cement extracts. The biocompatibility of MPCs has been extensively validated on bone cells and, according to the literature, they are not cytotoxic.^63,64^ In the prepared cements, concentrations above 0.5 M DAC in the composition, which corresponded to a release of citrate in the extract higher than 12 mM, appeared to be detrimental for cell viability, already at 24 h. For further long-term screening and comparison with reference cements, MPCs that appeared to be non-toxic at 24 h were exclusively selected. A TCP-based cement was used as a reference since its biocompatibility has been widely recognized.^65^ As a result, completely absent or very slight cytotoxic effects were found after cell exposure to the extracts of cements that released 2, 4, and 12 mM citrate. Furthermore, the long-term cytotoxicity of the selected cements was almost comparable to that of the TCP (within the range of more than 80% viability along the culture period). Of note, the higher the concentration of released citrate from the cements became, the greater was the observed cell viability, even at 72 h, thereby highlighting the beneficial effect of citrate in improving the cytocompatibility of the MPCs. In line with these data, a very recent study demonstrated that the citric acid-modified magnesium calcium phosphate cements, obtained by using the MgO, KH_2_P_2_O_4_, and Ca(H_2_PO_4_)_2_ particles, exhibited good cytocompatibility on osteoblast-like cells, indicating their potential application for bone regeneration.^39^

## 5 Conclusions

In summary, this work gives new insights into characterizing the effect of citrate on the properties of MPCs: the prepared cements display good handling and mechanical properties, and their setting times are suitable to clinical needs; in addition, upon contact with aqueous media, they are able to alkalinize them and to release citrate. The biocompatibility of some of the developed cements was also demonstrated, emphasizing the importance of finely tuning the concentration of citrate that is released from the material in biological fluids. The absence of cytotoxicity, the alkalizing properties of citrate, and its pivotal role in the bone tissue homeostasis, confer to this system the potential to properly prevent bone pathologies that are often related to acidosis, such as osteopenia and osteoporosis, and to favor the osteoinductive and osteoconductive properties of scaffolds intended for bone tissue engineering.

## Supporting Information Available

(i) UV spectra of DAC standard solutions and calibration line, (ii) photos of sample G after mixing and upon setting, (iii) XRD pattern of A and G, (iv) FE-SEM micrographs of samples, and (v) heat flow profile of TMP/water mixture and DAC (PDF)

## Abbreviations Used

CPCs: Calcium Phosphate Cements
MPCs: Magnesium Phosphate-based Cements
TMP: Tri-Magnesium Phosphate
DAHP: Di-Ammonium Hydrogen Phosphate
DAC: Di-Ammonium Citrate
P/L: Powder to Liquid (ratio)
TCP: α-tricalcium phosphate-based cement
XRD: X-rays Diffraction
PDF: Powder Diffraction File
FE-SEM: Field Emission Scanning Electron Microscopy
TGA/DSC: Thermogravimetry/Differential Scanning Calorimetry
IMDM: Iscove’s Modified Dulbecco’s Medium
FBS: Fetal Bovine Serum
