# Supplementary material for: Unravelling the Effect of Citrate on the Features and Biocompatibility of Magnesium Phosphate-Based Bone Cements
Source: ACS Biomater Sci Eng. 2020 Sep 9;6(10):5538–48. doi: 10.1021/acsbiomaterials.0c00983 (PMC8011797; doi:10.1021/acsbiomaterials.0c00983)
Supplement: Supplementary file 1 — ab0c00983_si_001.pdf [file ab0c00983_si_001.pdf]

Supporting Information

Unravelling the effect of citrate on the features and biocompatibility of magnesium phosphate-based bone cements

*Rita Gelli[a], Gemma Di Pompo[b], Gabriela Graziani[b], Sofia Avnet[b], Nicola Baldini[b,c], Piero Baglioni[a], Francesca Ridi[a]**

a) Department of Chemistry "Ugo Schiff" and CSGI, University of Florence, via della Lastruccia 3-13, 50019 Sesto Fiorentino (FI), Italy

b) Orthopaedic Pathophysiology and Regenerative Medicine Unit, IRCCS Istituto Ortopedico Rizzoli, via di Barbiano 1/10, 40136 Bologna, Italy

c) Department of Biomedical and Neuromotor Sciences, University of Bologna, 40127 Bologna, Italy

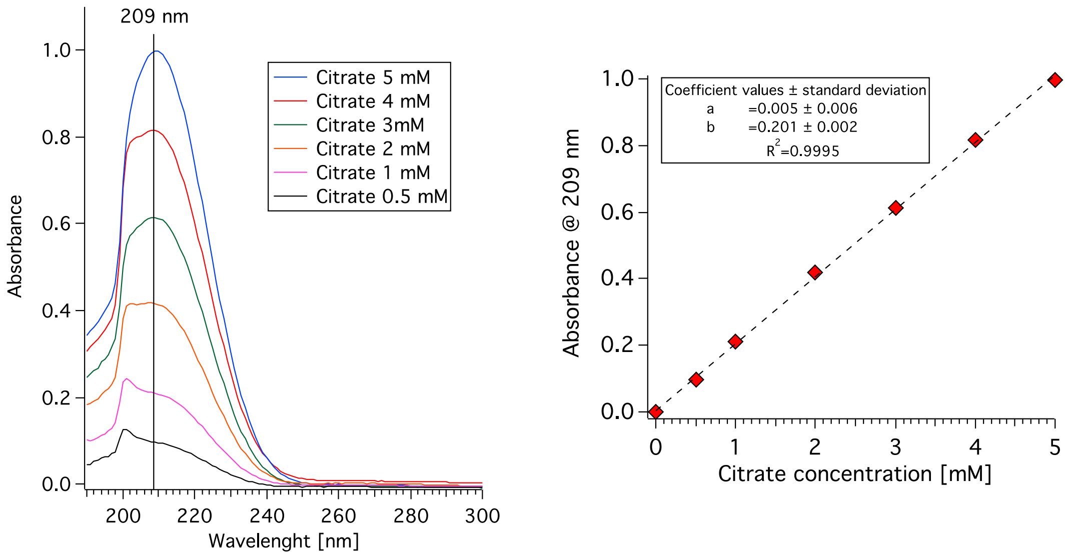

(a) (b)

Figure S1. (a) UV spectra of DAC standard solutions; (b) calibration line (y=0.201x+0.005).

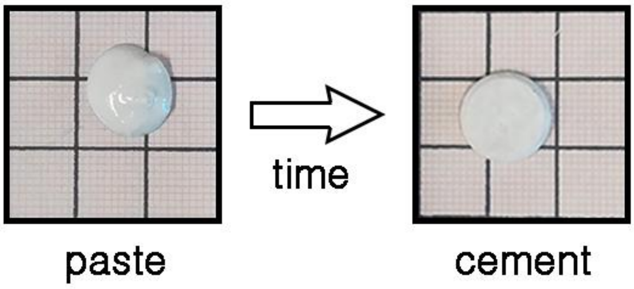

Figure S2. Photo of sample G immediately after mixing (left) and after setting in a mold for 5 days (right).

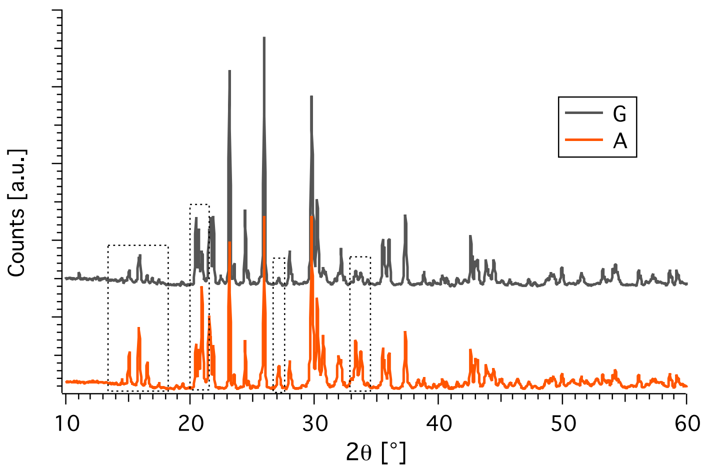

Figure S3. Direct comparison of the XRD pattern of cement A (no citrate) and cement G (DAC 2.0 M/DAHP 1.5 M). The highlighted areas show the decrease in struvite diagnostic peaks in sample G.

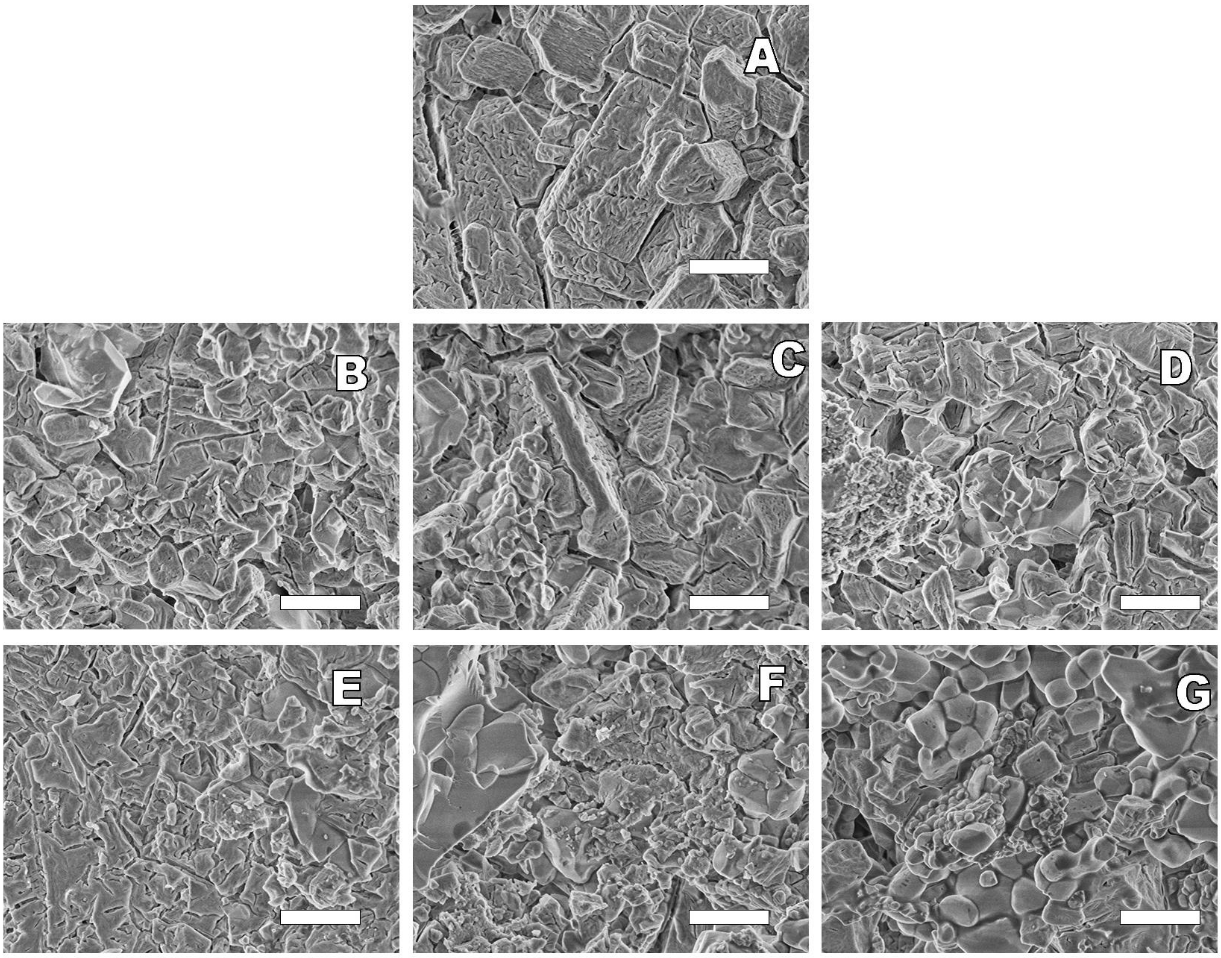

Figure S4. FE-SEM micrographs of the investigated cements, which reveal the heterogeneous morphology of the material. The identity of each sample is reported on the picture. Scale bar: 2 µm.

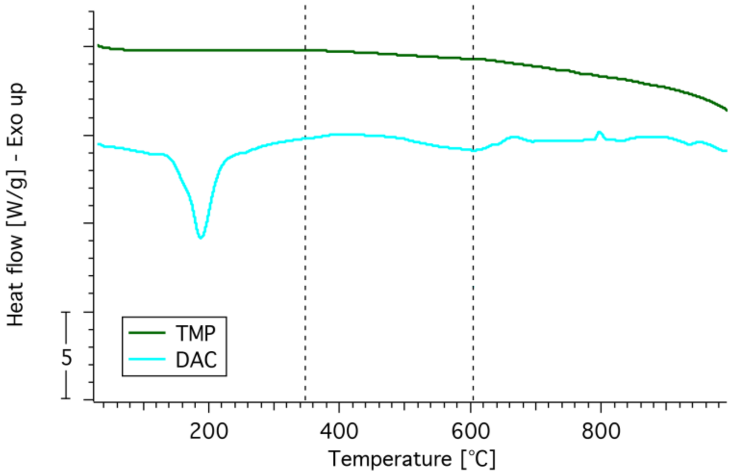

Figure S5. Heat flow profile of a mixture of TMP and water (green curve) and di-ammonium citrate (blue curve), which do not show any peak in the region of interest.
